# Evaluation of the current knowledge limitations in breast cancer research: a gap analysis

**DOI:** 10.1186/bcr1983

**Published:** 2008-03-27

**Authors:** Alastair Thompson, Keith Brennan, Angela Cox, Julia Gee, Diana Harcourt, Adrian Harris, Michelle Harvie, Ingunn Holen, Anthony Howell, Robert Nicholson, Michael Steel, Charles Streuli, Breast Cancer Campaign Gap Analysis Meeting (2 November 2006, London, UK)

**Affiliations:** 1 Department of Surgery and Molecular Oncology, University of Dundee, Ninewells Avenue, Dundee DD1 9SY, UK; 2 Wellcome Trust Centre for Cell Matrix Research, Faculty of Life Sciences, University of Manchester, Oxford Road, Manchester M13 9PT, UK; 3 Institute for Cancer Studies, University of Sheffield Medical School, Beech Hill Road, Sheffield S10 2RX, UK; 4 Tenovus Centre for Cancer Research, Welsh School of Pharmacy, Cardiff University, Redwood Building, King Edward VII Avenue, Cardiff CF10 3NB, UK; 5 The Centre for Appearance Research, School of Psychology University of the West of England, Frenchay Campus, Coldharbour Lane, Bristol BS16 1QY, UK; 6 Cancer Research UK Molecular Oncology Laboratories, Weatherall Institute of Molecular Medicine, John Radcliffe Hospital, University of Oxford, Headley Way, Headington, Oxford OX3 9DS, UK; 7 Family History Clinic, Nightingale & Genesis Prevention Centre, Wythenshawe Hospital, Southmoor Road, Manchester M23 9LT, UK; 8 Academic Unit of Clinical Oncology, School of Medicine and Biomedical Sciences, University of Sheffield, Beech Hill Road, Sheffield S10 2RX, UK; 9 Breast Cancer Prevention Centre, South Manchester University Hospitals NHS Trust, Wilmslow Road, Manchester M20 4BX, UK; 10 University of St Andrews, Bute Medical School, University of St Andrews, Fife KT16 9TS, UK

## Abstract

**Background:**

A gap analysis was conducted to determine which areas of breast cancer research, if targeted by researchers and funding bodies, could produce the greatest impact on patients.

**Methods:**

Fifty-six Breast Cancer Campaign grant holders and prominent UK breast cancer researchers participated in a gap analysis of current breast cancer research. Before, during and following the meeting, groups in seven key research areas participated in cycles of presentation, literature review and discussion. Summary papers were prepared by each group and collated into this position paper highlighting the research gaps, with recommendations for action.

**Results:**

Gaps were identified in all seven themes. General barriers to progress were lack of financial and practical resources, and poor collaboration between disciplines. Critical gaps in each theme included: (1) genetics (knowledge of genetic changes, their effects and interactions); (2) initiation of breast cancer (how developmental signalling pathways cause ductal elongation and branching at the cellular level and influence stem cell dynamics, and how their disruption initiates tumour formation); (3) progression of breast cancer (deciphering the intracellular and extracellular regulators of early progression, tumour growth, angiogenesis and metastasis); (4) therapies and targets (understanding who develops advanced disease); (5) disease markers (incorporating intelligent trial design into all studies to ensure new treatments are tested in patient groups stratified using biomarkers); (6) prevention (strategies to prevent oestrogen-receptor negative tumours and the long-term effects of chemoprevention for oestrogen-receptor positive tumours); (7) psychosocial aspects of cancer (the use of appropriate psychosocial interventions, and the personal impact of all stages of the disease among patients from a range of ethnic and demographic backgrounds).

**Conclusion:**

Through recommendations to address these gaps with future research, the long-term benefits to patients will include: better estimation of risk in families with breast cancer and strategies to reduce risk; better prediction of drug response and patient prognosis; improved tailoring of treatments to patient subgroups and development of new therapeutic approaches; earlier initiation of treatment; more effective use of resources for screening populations; and an enhanced experience for people with or at risk of breast cancer and their families. The challenge to funding bodies and researchers in all disciplines is to focus on these gaps and to drive advances in knowledge into improvements in patient care.

## Introduction

Significant advances in the prevention, diagnosis and management of breast cancer have been made in recent years based on the clinical application of scientific discoveries. However, breast cancer remains a complex disease process affecting millions worldwide, and further advances in scientific knowledge and clinical care could improve many lives. It is timely to review the current position of breast cancer research because funding bodies, researchers and clinicians work in an exciting age of discovery but have limited resources.

In November 2006, the research charity Breast Cancer Campaign convened a panel of leading breast cancer researchers, as an initial event, to debate and identify the limitations of current research into the pathophysiology, detection, treatment, prevention and psychosocial aspects of breast cancer. The aims of this analysis were as follows: To determine the gaps in our knowledge of breast cancer that, if resolved, could result in benefits to patients; To encourage breast cancer researchers and funding bodies worldwide to focus their resources on the highlighted areas of research to achieve a substantive impact for patients; To make recommendations for priority action.

This gap analysis represents a unique insight into breast cancer research in the UK and the challenges involved in directing efforts to areas in need of further investigation likely to result in advances in the management of breast cancer.

## Materials and methods

Current and former members of the Breast Cancer Campaign Scientific Advisory Board leading scientists and clinicians resident in the UK were invited to participate in the gap analysis meeting. The choice of participants was based on publication record, research activity and clinical stature, and selected using a database of researchers developed since the inception of the Breast Cancer Campaign in 1988.

Seven key research areas were selected for review in the gap analysis by the Breast Cancer Campaign and the Scientific Advisory Board, taking into account UK, European and USA themes in scientific meetings focused on breast cancer and UK Government analyses of research funding streams: Genetics of breast cancer; Initiation of breast cancer; Progression of breast cancer; Therapies and targets in breast cancer; Disease markers in breast cancer; Prevention of breast cancer; Psychosocial aspects of breast cancer.

Prior to the event, participants were asked to review relevant literature and construct short presentations summarising their areas of expertise and identifying potential research gaps. Key participants had already conducted, published and/or reviewed systematic evidence, literature reviews and evidence-based guidelines. As a result they were considered to be opinion leaders in their field. Further, additional, systematic literature reviews for each of the seven areas under consideration was not performed.

For each theme, 6 to 10 UK breast cancer researchers of national or international standing in their fields of research were invited and accepted participation in the event. Twenty-three invitees declined to contribute to the gap analysis.

On 2 November 2006 a one-day meeting was convened in London, UK. In the initial subgroup sessions, each participant gave a presentation to their group on pre-agreed topics relevant to the gap analysis for their assigned breast cancer research area on which they were experts. There were constructive debates of the content and the issues raised by the presentations were explored further. Breast Cancer Campaign staff members acted as facilitators throughout the analysis process.

Issues explored during the gap analysis were structured around the following questions: What do we already know; What are the gaps in our knowledge; What are the problems that need to be overcome to fill these gaps; What are the translational implications?

After collating the information resulting from these discussions for each of the seven themes, this four-point structure was used to present the content of each theme to the other participants and to discuss their findings in an open forum.

This iterative process continued as evidence-based expert opinion from the one-day meeting was cross-referenced, shaped and developed during subsequent weeks. Each group formulated a summary paper for their research area, incorporating key references, which was then circulated to the participants of the respective groups for further refinement. These seven themes were collated into a unified position paper, which is what we present here.

## Results and discussion

### 1. Genetics of breast cancer

A summary of the gap analysis for the genetics of breast cancer is given in Table 1.

**Table 1:** Summary of the gap analysis for the genetics of breast cancer

What do we know?	Multiple genes of different penetrance are involved in the predisposition to breast cancer.
	Genome wide screens and somatic genetic approaches are identifying further genes involved in breast cancer.
What are the gaps?	Detailed understanding of the actions of BRCA1 and BRCA2.
	Knowledge of large-scale genetic rearrangements in tumour cells.
	The important variants, effects and interactions of low-penetrance genes.
	Further identification of point mutations and epigenetic changes.
Problems	The quality, quantity and accessibility of materials.
	Funding for large-scale experiments (such as sequencing) using expensive equipment.
	Bioinformatic analysis skills.
Translational implications	Classifying breast tumours according to the signalling pathways that are disrupted to predict prognosis and response to therapy.
	Determining the relevance of somatic events to prognosis and response to therapy.
	Generate new, targeted therapies based on target discovery.
	Better genetic risk estimation.
Recommendations	Encourage development of research techniques to allow integrated analysis of sequence level, epigenetic and large-scale somatic changes.
	Engage in national initiatives for activities such as high-throughput re-sequencing and UK controls.
	Encourage research involving intermediate phenotypes.

#### What do we know?

Several genes bearing high-penetrance mutations have been implicated in inherited predisposition to breast cancer, the most important of these being in the *BRCA1 *and *BRCA2 *genes. However, *BRCA1 *and *BRCA2 *account for less than 5% of all breast cancer and, in recent years, breast cancer susceptibility genes other than *BRCA1 *and *BRCA2 *have been identified. These genes fall into two broad categories: Those containing rare moderate-penetrance alleles such as *CHEK2*, *ATM *and *BRIP1 *[1-3]; Those carrying more common low-penetrance alleles [4].

Large genome-wide association and candidate gene studies to identify the latter are just beginning to bear fruit [5-8].

Intermediate phenotypes such as radiosensitivity and mammographic density have quite strong genetic components and further study of these may provide some insight into novel breast cancer susceptibility genes.

Since the breast cancer genes *BRCA1 *and *BRCA2 *were cloned in 1994 and 1995, respectively, research efforts have concentrated on developing an understanding of the cellular functions of the large multidomain proteins encoded by *BRCA1 *and *BRCA2 *and the mechanisms by which loss of their functions causes breast cancer. An understanding of these mechanisms is relevant not only to families with *BRCA1 *and *BRCA2 *mutation carriers but also in sporadic breast cancer, in which the same or related genetic pathways may also be aberrant.

Cells deficient in BRCA1 and BRCA2 are extremely sensitive to DNA-damaging agents and are defective in repairing DNA double-strand breaks by homologous recombination, being impaired in the recruitment and filament formation of the recombination protein RAD51 [9,10]. More recently, new functions have been identified for both proteins: BRCA1 and its partner BARD1 form an E3-ubiquitin ligase that is recruited to sites of DNA damage and activated by the DNA damage checkpoint, promoting ubiquitylation [11]. The first *in vivo *substrate for such ubiquitylation events has been identified as CtIP [12].

An important development in recent years has been the identification of the links between the BRCA1 and BRCA2 pathways and proteins involved in Fanconi anaemia. Several genes cause Fanconi anaemia, and most of these encode proteins that are involved in a complex that ubiquitylates FANCD2 at K561. The *FANCD1*, *FANCJ *and *FANCN *gene products act downstream of this step. *FANCD1 *was found to be the *BRCA2 *gene [13]. *FANCJ *encodes BRIP1, which interacts with the BRCT domain of BRCA1 and, of note, mutations in this gene can also cause breast cancer [3]. Lastly, *FANCN *encodes PALB2, which interacts with BRCA2, and is also a breast cancer susceptibility gene [14].

#### What are the gaps?

There remain gaps in our knowledge of cancer predisposition genes, both in identifying genes responsible for low-penetrance disease and the interactions with environmental factors. Increasing knowledge of BRCA1 and BRCA2 acts as an exemplar resulting in improved patient care. However, deficiencies remain in our understanding of how BRCA1 or BRCA2 dysfunction causes breast cancer, for example, it is unclear why BRCA1 deficiency is associated with triple-negative (basal-like) breast cancer. We also need to find other proteins that interact with BRCA1 and BRCA2 and elucidate the post-translational modifications that occur as a result of these interactions. For example, the functional consequences of CtIP ubiquitylation and its implications for RAD51 recruitment are not yet known; furthermore, it is likely that there are other substrates for BRCA1/BARD1.

Only a relatively small proportion of breast cancers are caused by the loss of BRCA1 or BRCA2 function; most arise as a result of somatic mutation or changes in expression of a number of other genes. The list of genes showing somatic point mutations in breast cancers is beginning to be identified using genome-wide sequencing approaches [15]. Epigenetic changes such as DNA methylation and histone modification can cause loss or gain of gene expression, and genome-wide screens for these are being actively pursued. More work is needed in both of these areas.

To date, little progress has been made towards cataloguing larger-scale genetic rearrangements, such as translocations, deletions and amplifications, which occur frequently and are the hallmarks of tumour cells and have been particularly useful in the haematological malignancies. These changes also need to be related to tumour subtype. Reciprocal translocations seem to be common; for example, one translocation causes abnormal expression of the NRG1 gene which encodes ligands of the epidermal growth factor receptor (EGFR) family [16]. Work has begun to characterise and catalogue these events using high-resolution DNA microarrays. In the future it will be important to devise approaches to examine sequence-level, epigenetic and large-scale changes together and relate these to clinical features to form a complete and integrated picture.

As common, low-penetrance alleles continue to be identified, future challenges lie in identifying the causative variants within the haplotype blocks containing the associated marker single nucleotide polymorphism (SNP). First, this requires high-throughput sequencing capacity to detect all common variants in haplotype blocks spanning typically 30 to 150 kb, in at least 48 people. Second, case and control DNA collections from non-European populations are needed to separate alleles that are completely correlated in European populations. Third, sensitive biological assays need to be developed to determine the differences in function of the potentially causative alleles. It will be important to encourage a high degree of collaboration between research groups, in particular between epidemiology and basic biology. Furthermore, these studies would benefit from shared resources including first-class sample collections, such as a UK national control set.

A potentially useful but currently under-developed approach is to examine other phenotypes linked with breast cancer that are themselves determined genetically. These include mammographic density, radiation sensitivity, cell migration and circulating levels of hormones and growth factors.

#### What are the problems?

The number of patients required for gene searches requires large national and international consortia. The quality, quantity and form of the clinical material (blood derivatives, frozen tissue, formalin fixed tissues), and the handling or products from these present a significant challenge. The increasing sophistication of equipment and the level of technical expertise are reflected in the need to integrate the data in a meaningful way presenting a substantial bioinformatics challenge. Techniques for high-throughput re-sequencing are being developed, but funding is needed to make these accessible. Thus, this type of research is particularly resource intensive and requires a high level of collaboration.

#### Translational implications

Therapies based on developing an understanding of the role of BRCA1 and BRCA2 in DNA repair are already in clinical trials (including cisplatin and PARP inhibitors), and improving our understanding of the many functions of BRCA1 and BRCA2 will no doubt generate further targets for therapy. Increasing efforts to understand genetic events should allow us to perform the following functions: Classify breast tumours according to the signalling pathways that are disrupted and to predict prognosis and response to therapy; Determine the relevance of somatic events to prognosis and response to therapy; Generate new, targeted therapies based on target discovery.

Identifying combinations of inherited variants that predispose to breast cancer will allow us to better estimate risk in families with breast cancer and help to characterise defective signalling pathways. DNA collections from cancer prevention trials and clinical trials of radiotherapy and chemotherapy are underway to relate DNA variants to treatment response. While the trial populations of the UK, Europe and the US may provide complimentary data, international exchanges of such information should enhance future patient management.

### 2. Initiation of Breast Cancer

A summary of the gap analysis for the initiation of breast cancer is given in Table 2.

**Table 2:** Summary of the gap analysis for the initiation of breast cancer

What do we know?	Animal models have given us great insight into the molecular pathways involved in breast development and dysregulation in cancer.
What are the gaps?	The relationship of signalling pathways to ductal and acinar breast architecture.
	The need for widespread use of more appropriate *in vivo *and culture methods.
	The importance of stroma and other cell types, cell adhesion and the extracellular matrix.
	Understanding stem cells.
	Understanding mechanisms of epithelial apoptosis.
	Understanding how pregnancy and functional differentiation in the breast protect against breast cancer.
Problems	The breast cell lines used and their culture conditions.
	A wider variety of promoters with spatial, temporal and differentiation control of gene expression is needed.
	The need for mouse models of specific breast cancer types, for example, triple negative breast cancer.
	The implantation methods for single cells *in vivo*.
Translational implications	Understanding the complex interactions between cell types should provide new opportunities for intervention.
	Identifying pre-invasive changes has implications for patient-tailored approaches.
Recommendations	Develop three-dimensional cell culture models, containing multiple cell types, which reflect the tissue architecture of the normal and diseased breast.
	Generate better animal models, in which gene expression can be manipulated in each cell type of the mammary gland and will not be altered by transdifferentiation or dedifferentiation.
	Gain a greater understanding of the genetic changes that occur within atypias and DCIS.

To decipher the molecular basis of the initiation and progression of breast cancer, it is critical that we fully understand the key features and genes involved in normal mammary development. Changes to developmental processes may lead to tumour initiation and the influences of endocrine agents, growth factors and environmental carcinogens on normal and developing breast components is largely unknown,

#### What do we know?

Significant progress has been made in determining the local factors that control all stages of mammary development, largely through the generation of an extensive array of transgenic and knockout mouse strains [17]. Such animal models are complemented by classic embryological approaches that enable the transplantation of a complete mammary gland, ductal rudiments or, more recently, stem cells into cleared fat pads [18-20]. These approaches have imparted considerable knowledge about a wide array of developmental signalling pathways that are now known to be dysregulated in tumours. For example, amphiregulin/EGFR signalling is required for the branching and outgrowth of the ductal epithelial tree during pubertal development [21,22], while overexpression of both the ligand and receptor, and the related receptor ErbB2, is associated with poor prognosis in breast cancer [23]. Other examples include the IGF, integrin, Notch, NF-κB, STAT, TGF-β and Wnt pathways [24-29].

#### What are the gaps?

##### Tissue architecture

Many of the signalling pathways controlling normal mammary development have been identified and the genetic circuit diagrams that link the different pathways are emerging [17]. However, it is not clear how these signals cause ductal elongation or branching at the cellular level, or even how they maintain normal ductal or acinar architecture. Although analysis has focused on epithelial cells, the stromal, endothelial and immune components are also crucial for development [30]. For example, fibroblasts and macrophages are required for ductal growth [31]. However, many of the stroma-derived signals are poorly understood, as is the reciprocal communication between the epithelium and stroma and the signalling pathways controlled by the interaction between luminal and myoepithelial cells.

In addition, the importance of cell adhesion and the extracellular matrix has been underestimated, although it is becoming increasingly clear that both adhesion and matrix-derived signals modify many signalling pathways and provide a spatial checkpoint for developmental decisions [32]. Critically, changes in cell adhesion, through matrix remodelling or altered adhesion receptors, underpin both tissue disorganisation in early breast cancer and progression to malignancy [33,34], but their involvement in tumour initiation is not well understood.

##### Stem cells

The recent development of technologies allowing enrichment of mammary gland stem cells has been a significant step forward [19,20,35]. However, it is still not possible to purify these cells to homogeneity, and we do not fully understand their location within the ducts or the mechanisms involved in their differentiation into luminal and myoepithelial cells [36,37]. Moreover, it is not certain whether cancer stem cells are derived from normal stem cells or an intermediate progenitor cell, or how they are influenced by stromal factors [38]. In fact, it is far from clear that tumours are derived from stem/progenitor cells at all, as opposed to reprogrammed differentiated cells, let alone whether differences between cancer stem cells might be responsible for the development of different tumour subtypes.

Although considerable progress has been made towards understanding the mechanisms controlling epithelial apoptosis in the mammary gland, we know little about the sensitivity of stem/progenitor cells to apoptosis signals [39,40]. Cells of the main ducts survive involution, while alveoli and terminal ducts are lost, although the reason for this difference is not clear.

We also have little understanding of how early pregnancy and functional differentiation of the breast protects against cancer, and whether this is related to stem cell dynamics [41].

Consequently, we urgently need more appropriate *in vivo *and culture models to resolve the mechanisms of both normal breast development and tumour initiation. Furthermore, use of these sophisticated models needs to become more widespread.

#### What are the problems?

##### Culture models

Breast cancer has traditionally been studied by cell and molecular biologists using long-lived cell lines that are derived from late stage tumours and which display few of the cellular properties of normal breast epithelial cells. In addition, we now appreciate that breast cancer involves growth in three dimensions and the contribution of various breast cell types. Furthermore, techniques for studying the initiation and progression of cancer are largely restricted to analysing the luminal epithelium. Recently there has been a shift from two-dimensional to three-dimensional culture models, which better reflect the tissue environment found *in vivo *[42,43]. However, most of these models still contain only luminal epithelial cells. In the future, culture models containing all mammary cell types, as well as those amenable to examining ductal branching, modelling the stem cell niche and even assessing whether an isolated cell is a stem cell, will provide fertile avenues for analysis [44].

Excellent imaging technologies are emerging to explore the dynamic nature of mammary gland development and neoplasia both in culture and *in vivo*; applying them to human cancer cells and ensuring their widespread use will be of enormous value [45,46].

##### Genetic analyses

Expression of transgenes or gene ablation specific to the mammary gland is usually achieved by placing the transgene or Cre recombinase under the control of a milk gene promoter or the MMTV promoter [47]. However, these promoters are active only in the luminal epithelium and, in the case of the milk protein genes, limited to a particular developmental stage. More recently, the keratin 5 promoter has been used to target myoepithelial and basal cells [48]. Despite this, a wider variety of promoters would improve the spatial and/or temporal control gene of expression. This will be helped considerably by generating an atlas of mammary gland development. Further refining markers to identify stem and progenitor cells, and fibroblasts, will pinpoint other valuable promoters. Greater use of inducible transgene systems, such as the Tet-On system, will allow transgene expression to be restricted to specific time intervals [49].

Another significant problem with existing promoters is that their expression in the luminal epithelium depends on its differentiation; activity is therefore lost if a transgene causes transdifferentiation or dedifferentiation within a tumour. The problem can be overcome through the development of 'hit and run' transgenics. These include lines where the expression of a transgene is under the control of a housekeeping gene but is prevented by a lox-stop-lox cassette. Excision of the stop codon by crossing with mice carrying a gland-specific Cre recombinase then leads to continuous and consistent transgene expression. These and similar sophisticated strategies will allow the more realistic activation of breast cancer oncogenes, and will provide better opportunities to understand how they cause disease within the correct tissue environment. It would also be valuable to produce a series of transgenic reporter gene mice equivalent to the TOPGAL strain, to monitor changes in developmental signalling pathways [50].

There will also be great benefit in generating better mouse models of specific types of human breast cancer, ranging from triple negative breast cancer (oestrogen receptor negative, progesterone receptor negative and HER2 negative) to oestrogen receptor positive tumours. Similarly, straightforward protocols for implanting and growing primary human cancer cells in the cleared mammary fat pad of immunocompromised mice are required. Significant progress has been made in generating such humanised models, but it is still not possible to implant individual cells [51].

#### Translational implications

Continuing to study normal breast development will provide many useful insights into the earliest stages of breast cancer initiation. Tumour development depends on signals between the stroma, myoepithelial cells and luminal cells, and therefore gaining a better understanding of how these cells communicate in normal mammary gland and early breast disease will provide new opportunities for intervention.

In addition, we know little about the genetic changes that occur in atypias, lobular or ductal carcinoma in situ (DCIS) [52], although they are most likely to be within components of the signalling pathways that control normal development. Producing timelines of these mutations for different breast cancer subtypes, similar to those generated for colon cancer [53], would be a major step forward. Molecular profiling of breast cancers has begun to classify tumours [54], and relating them to the TNM classification will be clinically valuable for biomarker analysis and therapy. Differences between profiles may represent alterations in specific signalling pathways, so mapping this information onto a timeline of when mutations occur in breast cancer will enable clinicians to tailor specific treatments to individuals.

### 3. Progression of Breast Cancer

A summary of the gap analysis for the progression of breast cancer is given in Table 3.

**Table 3:** Summary of the gap analysis for the progression of breast cancer

What do we know?	Oestrogen receptor, receptor tyrosine kinase (RTK) and DNA repair pathways have been researched extensively.
	Around 50% of DCIS will progress to invasive disease if untreated, with 12% to 20% recurring at 10 years despite appropriate treatment.
What are the gaps?	Understanding the complexities of breast cancer intracellular signal transduction pathways, paracrine pathways, invasion, angiogenesis and metastasis including relevance of these mechanisms to clinical progression.
	Whether there are inherently migratory stem cells or is metastatic capacity acquired.
	Understanding time-dependent progression events, notably dormancy and reactivation of micrometastasis, at particular secondary sites.
	Understanding the emerging relationship between therapeutic resistance and metastasis.
	Causative factors underlying recurrence of DCIS or progression to invasive disease
	Understanding the interplay between stroma, myoepithelial and epithelial components during early progression and interplay between tumour cells, stroma and the immune system in metastasis.
	The need for improved preclinical models of the influences of the microenvironment, site-specific metastasis and dormancy.
	*In vivo *imaging technologies to study the dynamics of metastasis and relate this to signalling mechanisms, as well as means to manipulate these mechanisms to evaluate targeting potential.
Problems	Appropriate clinical samples to evaluate biomarkers and cellular endpoints.
	Appropriate preclinical models and improved research reagents.
	Increasingly complex and multidisciplinary research infrastructure.
Translational implications	Identifying patients at increased risk of dissemination.
	Effectively predict therapeutic response with growth inhibitors.
	Improve selection of patients with DCIS for adjuvant radiotherapy or endocrine therapies.
	Identify cellular targets for developing new agents to target breast cancer progression effectively and selectively.
Recommendations	Improve preclinical models, research reagents and technologies (including imaging).
	Enhance access to appropriate clinical material, notably matched samples during progression and sequential samples obtained during treatments including new agents.
	Consider the genetic signature/specific genetic lesions when exploring progression biology and designing clinical trials.

#### Intracellular inputs

##### What do we know?

The oestrogen receptor, receptor tyrosine kinase (RTK) and DNA repair pathways are key research areas in understanding the intracellular inputs for growth and progression of invasive breast cancer.

It is now established that steroid hormone or oestrogen receptor alpha (ERα) input, a fundamental driver for the growth of many breast cancers, should not be considered independent of additional signalling networks. This nuclear ERα may interplay with ERβ and there is emerging evidence that membrane-localised ERα may also have a role. A web of RTK signalling also contributes to breast cancer growth and progression, and these pathways can interact with ER when present [55-57]. Previous experimental deciphering of ER and RTK pathways has provided proof of principal that useful biomarkers and therapies (for example, endocrine therapies, erbB and kinase inhibitors) can stem from concerted research in breast cancer growth signalling biology [58].

DNA damage response (DDR) pathways and mechanisms for mitotic chromosome segregation are also of interest when considering breast cancer growth, progression and selectivity. They can be subject to genetic alteration, associating with tumourigenesis for inherited (for example, BRCA proteins) and for sporadic (for example, Aurora A: 30% to 60% overexpression) breast cancer because they cause genetic instability that may permit secondary alteration, accelerating the development of cancer. Equally, there are major implications for therapeutic efficacy, for example, for poly(ADP-ribose) polymerase inhibitors and BRCA mutations, and also resistance to taxanes when Aurora A is amplified [59].

##### What are the gaps?

Research into ER and RTK signalling is intense. Further deciphering of the complexities of breast cancer signal transduction pathways, their regulation and interplay, including elucidating downstream gene effects, is important, not least because therapeutic resistance is a persistent problem for all therapies targeting known pathways.

There are also limitations in our ability to subsequently evaluate RTK and ER signalling aspects emerging from experimental studies in clinical disease and to rapidly translate findings into clinically useful biomarkers and targets for new drug development.

Furthermore, the breadth of DDR and mitotic regulator alterations underlying the pathogenesis of breast cancer and its subtypes, or the point at which DDR alterations might contribute to disease progression is unknown.

#### Extracellular inputs

##### What do we know?

Local paracrine pathways (for example, those involving TGFβs), cell invasion into the extracellular matrix and angiogenesis all contribute to cancer growth and metastasis. Selective inhibitors of anti-Src and anti-HGF/Met, for some of the identified pathways involved in metastasis and inhibition of angiogenesis (for example, by targeting vascular endothelial growth factor signalling) are already in clinical practice [60].

##### What are the gaps?

Substantial questions remain about the biology of angiogenesis and metastasis. While studies of aggressive breast cancer cells *in vitro *have allowed significant progress in understanding adhesion and migration through matrix, matrix degradation (including mechanisms of matrix metalloproteinases) and *in vitro *invasiveness, subsequent translational applications have proved limited.

We have yet to fully explore the breadth of potential positive and negative regulators of invasion and metastasis, their mechanisms and interplay, and the role of the interaction of tumour cells with the stromal microenvironment and immune system during metastasis. The propensity for cancer to metastasise, apparently selectively, to certain end organs is poorly understood. Whether human breast cancers contain inherently migratory stem cells or whether metastatic capacity is acquired, and also time-dependent progression events, notably dormancy and reactivation of micrometastasis at particular secondary sites, remain poorly defined.

Equally, little is known about the emerging relationship between resistance to conventional therapies (for example, endocrine therapies) and metastasis [61] or the degree of redundancy of invasive elements or angiogenic pathways that may contribute to therapeutic resistance to anti-invasive and anti-angiogenic approaches.

#### Ductal carcinoma in situ and very early progression in breast cancer

##### What do we know?

Around 50% of DCIS will progress to invasive disease if untreated, with 12% to 20% recurring locally at 10 years despite breast conserving surgery and radiotherapy. Links have been suggested between early progression and mechanistic alterations both within the epithelial cells and in the interplay with associated basement membrane, myoepithelial and stromal cells, and mechanical constraints [62,63]. HER2 positivity and HER4 negativity, and high levels of cyclo-oxygenase-2 (COX-2) may have some relevance to invasive recurrence [64].

##### What are the gaps?

There is still a substantial gap in our knowledge of the causative factors underlying progression of DCIS to invasive disease and, in general, there are no compelling biomarkers that can robustly predict invasive recurrence. We also lack biomarkers that can select for patients who might benefit from existing therapies, since ER and HER2 (and EGFR) status are not routinely measured in DCIS outside clinical trials and so treatment is currently based on morphological assessment. Equally, there are few targets in DCIS to subvert progression other than ER for ER positive DCIS, with ER-negative DCIS thus being particularly problematic.

Issues regarding the very early progression of breast cancer reflect those for invasive breast cancer. There are substantial gaps in our ability to perform biological investigation of the intracellular and extracellular factors (notably interplay among stroma, myoepithelial and epithelial components) underlying growth and progression of DCIS, to dynamically monitor early progression, and to subsequently manipulate implicated pathways to address their therapeutic potential.

##### What are the problems?

###### Improved preclinical models

We need to develop better preclinical models in order to more accurately reveal the mechanisms involved in growth and progression of breast cancer and to evaluate potential targets. This is particularly important when trying to interpret the mechanisms of metastasis because dissemination commonly occurs at inaccessible sites, making clinical research material extremely difficult to obtain. However, it is equally relevant if we are to build on findings made using *in vitro *monolayer culture studies for growth signalling mechanisms.

Models are needed to allow researchers to investigate more accurately the influence of the microenvironment, the impact of the immune system and site-specific metastasis, and time-dependent progression including the phenomenon of dormancy. *In vivo *models will be essential to study these areas, and researchers may also benefit from *in vitro *three-dimensional assays encompassing stromal components, matrix and tumour epithelial cells [65], as well as studies over extended culture time *in vitro *and associated animal models.

Increased use of genetically modified animal models will be valuable, as will genetic manipulation of individual targets (for example, using RNAi) alone or in combination with existing agents in model systems to address how mechanisms of growth and spread might be exploited to provide new targeted therapies. Studies of very early progression will also require improved models, notably use of *in vitro *cultures of primary or mammosphere DCIS (again extending to longer-term culture), and of human DCIS xenograft and transgenic (for example, MMTV/HER2) models.

Alongside targeting individual candidate elements, more speculative approaches are warranted to broadly screen for synthetic lethality, for example, using RNAi libraries. We also need to embrace fully *in vivo *imaging technologies to decipher the dynamics of metastasis (and equally of very early progression) at a cellular level and relate this to signalling mechanisms (for example, using *in vivo *fluorescent reporter assays).

Powerful real-time imaging studies in animal models are emerging which indicate that the motile phenotype may be transient and confined to a subpopulation of cells at the tumour periphery in disease metastasising *in vivo*. Such heterogeneity is not easily modelled with *in vitro *studies. These observations illustrate the power of imaging systems for deciphering breast cancer biology, where the data have implications for interpreting whole tumour microarray/proteomic profiles and clearly confirm that time-dependent study of metastasis at a cellular level is essential.

###### Appropriate clinical samples

Appropriate clinical samples are needed to translate experimental findings into useful predictive biomarkers, and to confirm that therapeutic strategies stemming from basic research are relevant in patients. Meaningful study will require, where possible, improved access to clinical material with parallel therapeutic response and survival data, encompassing tissue microarray (TMA) and full-section resources. Studies will benefit from improved access to clinical samples of primary, local recurrent, lymph node and distant metastatic (where accessible) breast cancer, ideally comparing matched samples from patients to track potential biomarkers of progression. Studies of very early progression will also need increased access to clinical DCIS material to verify the relevance of experimental associations with invasive recurrence, considering gene expression signatures both for DCIS and invasive disease, preferably from the same patient.

It is also important that researchers obtain material from sequential samples (with parallel outcome data), taken before and during treatment with conventional therapies, and from biologically directed innovative trials of targeted therapies. Clinical trial design should increasingly incorporate improved tissue collection and pathology support (which has often been considered as an afterthought). Experience indicates that neoadjuvant studies and, where possible, access to patient samples treated longer term will be particularly valuable.

Studying samples obtained during treatment with anti-invasive agents (or, indeed, any new agents) will allow researchers to evaluate the ability of biomarkers and cellular endpoints stemming from experimental studies to provide surrogates for drug response, an important aim in the absence of long-term outcome data. This could be achieved by examining circulating tumour cells and sampling lymph nodes during treatment. In addition, evaluation of anti-invasive agents could benefit significantly from improvements in tumour tissue imaging of functional reporters.

These needs for clinical tissue clearly represent a significant challenge, particularly given that research must comply with ethical and legal considerations. To be achievable, patient recruitment to trials must be improved and the collection and sharing of tissue resources must be coordinated. For DCIS studies, sequential clinical samples taken pre-operatively during treatment with currently available agents (endocrine therapies, erbB or COX-2 inhibitors) or with emerging new therapies, are needed to confirm mechanisms and predictive capacity, and so enhanced trial design and better recruitment of DCIS patients is also essential.

###### Consideration of gene profiles

Greater attention needs to be given to gene profiles and the impact of genetic lesions in malignant epithelial cells and in the stromal background in clinical trial design and during the selection of patients for therapy. Expression profiling at the mRNA and protein level has revealed several subtypes of breast cancer, and it is important that intracellular and extracellular processes of growth and progression are further explored in relation to these, both through models that aim to recapitulate each class and through representative clinical material.

The non-tumour content of biopsies should also be considered since evidence is emerging to suggest that this has a significant effect on gene expression profiles [66]. Equally, several studies have indicated the importance of considering specific lesions in relation to therapeutic response, for example, Aurora kinase overexpression and taxane resistance, BRCA2 loss and response to platinum-based therapy [67], topoisomerase II alpha amplification and epirubicin response [68]. Considering these parameters may not only improve patient stratification for more effective trial design, but may also allow smaller patient cohorts to be studied, if these are selected rationally for therapy according to the status of the drug target or molecular lesion.

###### Improved research reagents

Better research reagents are needed to continue to accurately define new pathways in experimental material and to study clinical samples to verify these pathways as biomarkers in relation to pathology and outcome.

Signalling mechanisms in clinical samples can be detected using immunohistochemistry, including the use of phospho-specific antibodies [69,70]. However, if associations are to be accurate and meaningful, this urgently requires assays that have a reproducible, sensitive and specific performance in such material, incorporating improved quality control as has been achieved for ERα and HER2 assays [71-73]. Technologies such as fluorescence resonance energy transfer (FRET) have the potential to measure interplay between elements (for example, receptor dimerisation) in such material. However, in all instances these methodologies will require consensus regarding evaluation which should also aim for quantitative analysis, for example, through image analysis. More accurate quantification may potentially incorporate luminescent quantum dots as an alternative approach to visualise tumour markers.

Research to reveal new biomarkers and drug targets in experimental and clinical material will also increasingly need to incorporate high-throughput gene profiling with microarrays and TMA validation, as well as proteomics. In these latter areas, quality control urgently needs to be addressed and bioinformatic capabilities must be significantly enhanced on a national level if we are to manage and meaningfully interpret the increasing volume of signalling data that will emerge in relation to cellular endpoints and clinical outcome.

###### Research infrastructure

Overcoming these various barriers has obvious implications for research infrastructure. Studies will need to be increasingly multidisciplinary if we are to identify relevant determinants of breast cancer growth and progression; for example, requiring the integration of multiple expression/signalling studies, bioinformatics, imaging technologies, improved *in vitro *and *in vivo *models, genetic manipulation and clinical examination. This will depend on a backbone of realistic supportive funding, not only to maintain core strategies and associated quality control, but to ensure access to new technologies to pursue innovative research avenues (for example, *in vivo *imaging, genetically engineered models and high-throughput genomic screening).

A critical mass of expert staffing is essential, including expanding the breast cancer research talent pool through improved research training and more clearly structured career development. The need for carefully collected and documented clinical tissue with serial biopsies taken during therapy with defined treatments, TMAs, samples from distant sites and local recurrences made available to investigators is key. Recent legislative changes have made this more difficult and both surgical and pathology support are needed at a senior level. Both specialities have suffered severe cutbacks; collaborative contributions by academics in these areas are important and deserve funding, in addition to supporting the highest quality peer-reviewed independent research.

Pathology training will be increasingly important as we expand our technical capabilities for investigating clinical material. Standardisation of antibodies and other reagents is needed to compare results between investigators. Research would also benefit from increased sharing of experimental and clinical resources. Finally, increased investigator-driven studies and collaboration with industry will be essential if we are to improve patient recruitment for clinical studies aimed at understanding biological factors driving selective response.

##### Translational implications

If research can be tailored to the diverse questions regarding the intracellular and extracellular processes underlying breast cancer growth and progression, this should link tumour mechanisms to disease classification and prognosis.

To some extent this process is underway through microarray studies, which have led to the classification of invasive breast cancer into four categories, luminal, basal, HER2/neu overexpressing and normal [74], and the development of various gene expression signatures that can predict outcome. However, we need to build on these studies: first, so we can identify earlier patients at increased risk of dissemination and therefore allow selection for anti-invasive therapy and, second, to provide robust biomarkers to effectively predict therapeutic response with growth inhibitors. Filling the gaps outlined for very early progression may improve selection of patients with DCIS for adjuvant radiotherapy or endocrine therapies, while avoiding unnecessary treatment in others.

Equally, it is likely that we will reveal cellular targets for developing new agents to target breast cancer progression effectively and selectively (as well as being able to measure the target pathways dynamically during therapy to monitor clinical efficacy of novel inhibitors and improve trial design). Together, successful research should allow therapy to be increasingly individualised and include combination strategies aiming at maximally subverting tumour resistance and disease progression, improving the outlook for patients.

### 4. Therapies and targets in breast cancer

A summary of the gap analysis for therapies and targets in breast cancer is given in Table 4.

**Table 4:** Summary of the gap analysis for the therapies and targets in breast cancer

What do we know?	The selective use of combinations of surgery, radiotherapy, chemotherapy, and biological therapies has improved patient survival in recent years.
	Not all therapies used are effective on all patients.
What are the gaps?	There is an incomplete understanding of the biology of breast cancer including the effects of compensatory signalling pathways responsible for drug resistance.
	We cannot determine who goes on to develop metastatic disease or drug-resistant cancers.
	Individualisation of therapies could be improved.
	The optimal duration of therapy is unclear for many drugs.
Problems	There are insufficient model systems for the complexity and diversity of breast cancer.
	The need to understand not only the cancer, but the tumour microenvironment and patient characteristics (including drug metabolism and immune mechanisms).
	Availability of clinical material is scarce, particularly from metastatic disease tissues.
	The neoadjuvant model could be used more effectively.
Translational implications	Patients could be selected for appropriate therapy more effectively.
	Enhanced understanding of the sequencing, combinations and duration of treatments.
Recommendations	Build resources through high-quality, uniform, multicentre collection of clinical material from breast cancer patients before and during treatment (including neoadjuvant studies), including samples of primary tumours as well as metastatic deposits.
	Develop methods for easy, reproducible monitoring of response to and development of resistance to therapy, as well as early disease progression.
	Increase research efforts into the role of the tumour microenvironment and the immune system in the development and treatment of breast cancer.

The treatment of breast cancer has improved over recent years and has led to an increased survival rate for patients with tumours confined to the breast. This is partly due to breast screening resulting in early diagnosis but also the appropriate selection for patients of the surgical approach, radiotherapy, chemotherapy regimen and more recent therapies.

#### What do we know?

The introduction of new therapeutic strategies, including newer adjuvant endocrine treatments, radiotherapy scheduling, chemotherapy combinations and novel agents such as trastuzumab, has contributed to the increase in disease-free and, in some cases, overall survival. However, breast cancer recurs, sometimes many years after diagnosis, and the treatment of metastatic disease remains palliative. Thus, not all therapies used are effective and a proportion of patients (perhaps a majority for some therapies) receive one or more treatments which either are not required or fail to stem the disease.

Selection of multimodality therapy for an individual patient by a multidisciplinary team is based on the extensive evidence base for individual and combination therapies summarised elsewhere.

#### What are the gaps?

##### Incomplete understanding of the biology of breast cancer

Our understanding of the many cellular and molecular processes involved in the development of breast cancer is still incomplete. This hampers the identification of new therapeutic targets as well as the optimal use of the targets we know about. We have limited knowledge of which signals drive breast cancer cell growth, and how they promote the invasive nature of the disease. In addition, the role of the surrounding healthy tissues in tumour development, both at the primary and metastatic sites, needs to be clarified.

Current thinking is that to eradicate cancer cells we may need a combination of therapies targeting the tumour cells, their microenvironment and, potentially, their blood supply.

##### We cannot determine who goes on to develop advanced disease

Despite our best efforts a proportion of patients will develop advanced disease, and we do not currently have reliable tools to predict who these patients are. By using tumour grade, pathological node status, tumour size and other pathology features, a number of models have been designed to assess the risk of patients developing metastatic disease including the Nottingham Prognostic Index and Adjuvant Online. However, there are no established molecular markers used in clinical practice to determine with certainty whether a breast tumour is likely to metastasise to other sites, and therefore no easy way of selecting patients at early stages of the disease that will require more intensive treatment to prevent tumour progression.

In addition, there are no simple, non-invasive methods available for detecting the early stages of tumour progression, and patients often present with relatively advanced (symptomatic) disease.

###### Insufficient knowledge to provide precise, individualised therapies

One of the main problems when treating breast cancer is to determine which patients will benefit from particular therapeutic strategies, ensuring optimal results for each individual patient. Not only is this key to achieving the best possible outcome for patients who are likely to respond to any given treatment, but also to avoid treating those who will not benefit.

A few targeted treatments are available (for example, endocrine treatments and trastuzumab) that rely on identifying a receptor present on the tumour cells. Understanding the biology of breast cancer better is likely to help us develop new anti-cancer agents that effectively target specific receptors present in only a subset of patients.

In addition to the biological characteristics of the tumours, each patient has an individual capacity to metabolise drugs. This leads to variations in drug half-life that may partly explain why the response rate varies between patients receiving identical treatments. For example, many commonly used anti-cancer agents (including cisplatin, doxorubicin, tamoxifen and etoposide) are metabolised in the liver by enzymes of the p450 group, and there are documented variations in the activity of these enzymes between individuals.

Finally, the optimisation and combination of the current therapies to fit individual patients is often based on a trial and error approach, rather than a clear understanding of the biology of the tumour.

###### How to decide when to stop treatment?

We lack suitable methods for the early determination of recurrence and treatment failure.

For most current therapies, there is little long-term data to support when it is safe to stop treatment. If patients experience no side-effects and are free from cancer they are likely to want to continue their therapies even in the absence of any proven benefit. There are no easy, non-invasive, reproducible methods available for routinely monitoring subclinical disease progression and response to adjuvant treatment, and we rely on patients to present with symptoms in order to establish whether the tumour has recurred.

###### Who will develop drug-resistant tumours?

The basis of drug resistance is not well understood, and as a consequence we have no reliable methods of predicting who will go on to develop resistance to the commonly used therapies. We do not know how to avoid a resistant phenotype developing, and is it not clear whether changes in the frequency of drug administration and/or length of treatment contribute to this process.

###### Incomplete understanding of the role of the immune system

We do not fully understand how best to use the immune system to our advantage in breast cancer treatment, either as a vaccine or in the form of immunotherapy. The lack of suitable model systems for studying the immune response, as well as the many fundamental differences between the species most commonly used (rodents) and humans have hampered progress in this area. In addition we also lack understanding of how tumour cells suppress the immune response to ensure their survival and growth.

###### How do anti-cancer treatments adversely affect cancer cells?

Experimentally, it is becoming apparent that treatment of breast cancer cells with endocrine therapies can rapidly activate alternative signal transduction pathways, which may limit the initial anti-tumour response, allow resistance to develop and, ultimately, encourage invasive behaviour [75]. For example, EGFR/HER2 signalling is triggered by anti-oestrogens in various hormone-responsive breast cancer models that maintain residual downstream kinase activity, proliferation and cell survival [76]. Interestingly, 'compensatory' induction of alternative signal transduction is a phenomenon shared by other types of anti-cancer therapy, including anti-growth factors, chemotherapy and radiotherapy [77-79].

The full breadth and cellular impact of such compensatory signalling is largely unknown in breast cancer. To fill this gap we need to use high-throughput discovery tools to profile multiple signalling pathways and to explore the concept in a broader panel of models reflective of the various breast cancer subtypes. In addition, although changes in some signalling elements (for example, HER2, activity of various kinases including MAPK, JNK and p38) have been reported to be in place by the time of relapse, the drug-induced concept is largely unexplored in patients. We need increased access to 'on therapy' clinical samples to rectify this.

##### What are the problems?

There are many reasons for these gaps in our knowledge, including the following: A lack of suitable model systems reflecting the complexity and diversity of breast cancer; Limited access to clinical material from patients before and, in particular, during treatment; A severe lack of material from metastatic deposits made available for studies of target hits and biological response to therapies.

The neoadjuvant model provides a window of opportunity where therapy can be tested *in vivo *in humans to assess the effects of an intervention. It allows biological evaluation of tumour markers and normal tissue responses by histological, biochemical, molecular, imaging or clinical techniques. However, neoadjuvant studies have not usually involved adequate numbers of patients for what can be intensive study and individual centres often recruit too few patients in specific groups to ensure meaningful analysis; a multicentre approach is required to ensure progress.

Identifying new therapeutic targets is hampered by our limited understanding of the role of the tumour microenvironment and interactions with cancer stem cells in the development and progression of breast cancer. In addition, we do not understand the mechanisms underlying the acquired resistance to anti-cancer therapies. There are too few studies across disciplines to increase our understanding of the role of the immune system, an area where there is a lack of appropriate model systems and insufficient high-quality studies carried out in humans. Not enough attention has been paid to how drug metabolism by individuals affects response to treatment. This important point is not considered in drug trials on an individual basis, or linked to measurements of response, but may partly explain why there is such variation between patients receiving identical treatments. As assays for drug metabolising cytochrome p450s become available (for example, 2D6 for tamoxifen metabolism), this may move into clinical practice.

These gaps may be filled by developing improved model systems that seek to reflect the complexity of the human disease, combined with increased efforts to design multicentre studies using clinical material collected and processed in a uniform way. Particular areas that we need to strengthen are the increased use of neoadjuvant studies, providing researchers with valuable clinical material from breast cancer patients during treatment in the form of repeated biopsies.

Few studies have involved investigations of metastatic deposits, so our understanding of the biological changes of the tumour cells as they adapt to new environments is limited. Not many patients will undergo procedures that allow the collection of material from metastatic sites, and no single centre is likely to be able to collect significant numbers of quality specimens for research. As building these types of 'biorepositories' for future research is likely to take many years to accumulate numbers that allow meaningful data analysis, this requires a collaborative, long-term approach for which funding may be difficult to obtain (most funding bodies operate on a three- to five-year timescale before results are expected).

We therefore see an urgent need for high-quality, comprehensive, longitudinal sample collections (tumour, DNA, serum, plasma, urine) from breast cancer patients, coupled with extensive clinical information. Where appropriate, collaborations with researchers in other fields (for example, immunologists working in rheumatology or auto-immune diseases) are needed.

##### Translational implications

If we could fill the gaps identified here we anticipate that we would be able to select patients for appropriate therapy more accurately, start patients on therapies earlier and monitor progression and response. We would have a better understanding of how to sequence and combine therapies, an increased capacity to develop innovative and immunologically based therapies that subsequently prove suitable for treating the disease. For example, proof of principle experimental data reveal that intelligent targeting of induced signalling alongside the primary therapy can achieve a previously unobtainable level of cancer cell kill and substantially improve anti-tumour response [76]. If the drug-induced concept is reproduced *in vivo*, rationally designed combination strategies could have the potential to improve initial response and delay resistance and progression; this would have a positive impact on breast cancer survival rates. We would then be better placed to develop drugs with an improved therapeutic window and fewer side-effects, and be able to improve breast cancer survival rates.

### 5. Disease Markers in Breast Cancer

A summary of the gap analysis for disease markers in breast cancer is given in Table 5.

**Table 5:** Summary of the gap analysis for disease markers in breast cancer

What do we know?	Patient groups can be successfully stratified in clinical trials using biomarkers.
What are the gaps?	Optimum protocols for pathological assessment of DCIS and sentinel lymph nodes.
	Combining clinical, radiological, pathological and genomic data in trial populations.
	No robust validated markers have yet been developed for predicting response to chemotherapy or radiotherapy.
	There is no consensus for markers indicative of resistance to therapy.
	There is a need for improved prognostic indices based on disease markers.
Problems	New assays must be robust and reproducible.
	There is a need for standardisation of tissue handling.
	The impact of legislation, industrial involvement and academic pressures.
	Networks of collaboration employing systems biology are required.
Translational implications	Accurate recognition of the diversity of breast cancer.
	Identification of patients most likely to benefit.
	Identification of patients least likely to benefit from therapy and hence able to avoid toxicity.
Recommendations	Design innovative trials and translational studies to develop and evaluate predictive and prognostic markers.
	Develop close multidisciplinary collaboration with high-quality histopathology and rigorous scientific assessments to validate new markers important for patient outcome.
	Identify robust markers of resistance or sensitivity to therapy that can be applied across the spectrum of breast disease from screen-detected to metastatic breast cancer.

Developing new treatments for breast cancer and refining existing regimens are clearly important and exciting areas of research. The challenge is to ensure that new therapies reach the patients who will benefit most, and to identify patients for whom the harms outweigh the benefits or for whom the treatment will be ineffective [80]. To achieve these aims we need validated predictive and prognostic markers.

#### What do we know?

The gold standard for comparing new markers is testing against high-quality pathological assessment of tumour type, size, grade and lymph node stage.

Only two markers have been established so far in the routine assessment of breast cancer: ER (for predicting response to endocrine therapies) [80]; HER2 (for predicting response to trastuzumab) [80,81].

Although theses markers for predicting the response to endocrine and biological therapies are already available, even ER and HER2 are far from perfect; for example, assessment of HER2 status will still include some non-responding patients [81].

Intelligent trial design involving multidisciplinary teams is essential to ensure new treatments are tested in patient groups stratified using biomarkers. Large-scale studies of invasive breast cancer that have and are using these principles successfully include the trials of trastuzumab after adjuvant chemotherapy in HER2-positive breast cancer [81]. Biomarker-based trials may also be used to assess treatments for advanced breast cancer, but may be confounded by prior exposure of such cancers to multiple therapies.

Ideally, however, pre-operative (neoadjuvant) studies are required, using clinically relevant models and crossover designs to provide early evidence of a therapeutic effect and to differentiate responsive from non-responsive groups of patients [82].

#### What are the gaps?

##### Innovative trial and study design

Disease marker concepts should be applied to trials of treatments for pre-invasive disease including DCIS and to models of sentinel lymph node assessment, where funding is limited and where long-term follow-up is required to obtain robust clinical data, but where we need a better understanding of the pathophysiological processes involved. Two areas we need to address are as follows: The level of sentinel lymph node involvement that has a clinical impact; Optimum protocols for pathological assessment and systems for reproducibly categorising clinically relevant pathological metastatic disease [83].

Additional challenges include recognising the differences between laboratory studies and *in vivo *studies in humans where interactions between tumour and stroma, three-dimensional effects and vascularisation become relevant. Furthermore, combining clinical, radiological, pathological and genomic data in trial populations with innovative trial designs (such as in the MINDACT trial [84]) will allow us to relate, compare and combine established markers to, and with, new technologies in a range of settings. Success depends on close multidisciplinary collaboration at an early stage, as well as the highest quality histopathological and scientific evaluation.

##### Validating new markers

New markers may be best validated in trials of neoadjuvant or adjuvant therapies and in advanced disease, where clinical data and outcomes are robust and statistically significant. However, large numbers of patients may be required for small incremental differences in outcome.

The key question is: does introducing a new marker change clinical practice and therefore patient outcome? In the past, researchers have not paid enough attention to experimental design when assessing this; consequently the results have not always been standardised, reproducible or robust enough to apply to clinical practice. Developing rigorously controlled reagents, technical methods and appropriate interpretation requires adequate resources and to date this has rarely been forthcoming.

While ER-negative patients rarely respond to hormone therapies, a proportion of those classified as having ER-positive disease will also not respond [85]. No robust validated markers have yet been developed for predicting response to chemotherapy or radiotherapy. Many markers are favoured by local enthusiasts (for example, progesterone receptor [PgR], PS2 and cathepsin D) but we need high-quality clinical evidence to support their more widespread use.

Markers indicative of resistance to therapy (for example, ER-positive, PgR-negative tamoxifen-resistant cancers) have been proposed, but there is little agreement about methodology or cut-offs of scores for clinical application, or indeed their overall value. In addition, some markers may not be useful once regimens or therapies are superseded. We therefore need to compare, and potentially combine, markers such as the ER and PgR with pathological markers (such as histological type, grade and node metastasis), which have prognostic importance. Funding for robust studies evaluating these markers is crucial, but is rarely achieved without financial support from the pharmaceutical industry.

Researchers have identified validated intermediate endpoints, such as the effects on apoptosis, proliferation and ER down-regulation (for example, with fulvestrant therapy). However, further work is urgently needed using tumour pathology (for example, lymphovascular invasion and microstaging) and molecular biology, and exploring the potential of disease response markers, such as serum proteomic markers and circulating tumour cells. Subclass-specific markers based on microarray approaches have been identified and validated by immunohistochemistry [86,87], but have yet to be applied in clinical trials and clinical practice.

Therefore, although breast cancer can be classified according to histology and expression of RNA and protein, for example, into basal, HER2, luminal A and luminal B and normal breast-like subtypes [86,87], we need to develop this further to predict the prognosis of each subtype and the likelihood of response to therapies.

A further challenge is in understanding the complex factors influencing prognosis and in improving the quality control of reagents used in new technologies (such as proteomics, phosphoproteomics, epigenetics and assays of microRNA) so that they can be effectively applied to routinely available clinical material. In particular, integrating old and new methods and combining techniques (such as TMA, immunohistochemistry, fluorescence *in situ *hybridisation, data storage and analytical methods) may allow us to develop new composite prognostic indices. A significant challenge to the development and validation of predictive markers is counteracting the effects of storing tumours and blood derivatives on proteins (including phosphorylated proteins) and in proteomic studies. We clearly need careful research into these processes and how to take them into account.

#### What are the problems?

Any new assays that are developed must stand up to the day-to-day challenges of clinical practice. The quality of service delivered in routine clinical practice varies, even for basic markers such as ER and HER2 [88]. RNA-dependent assays have been considered less robust than protein- or DNA-based assays in the breast cancer setting. However, RNA-based polymerase chain reaction (PCR) technology may well become a standard of care as an intra-operative detection method for tumour in sentinel lymph nodes. Techniques must undergo regular quality assurance both during development (in the research setting) and in subsequent laboratory and clinical use; marker validation requires time and resources and subsequently convincing the professions to apply them.

Implementing advances in molecular understanding may be limited to paraffin tissues as a source of standardised and stable processed material in the foreseeable future. Advances rely on having appropriate technologies and tissues available and on training for scientists and clinicians. The timing and quality of fixation methods, tissue handling and preparation are critical both in trials and in routine clinical use. Clinical trials have rarely developed standard procedures for collecting and documenting tissue, although more recent innovative trials do so [84]. Even then, delivery time and costs may prevent techniques and markers from being widely used in clinical practice.

Smaller cancers, often detected by breast screening, present particular problems. Not only may there be little material for studying the primary tumour, but lymph node micrometastases are more likely to be present than larger deposits and may change the classification of nodal status. Such patients may still have an excellent prognosis, although conventional indices relating node involvement to disease behaviour may result in adjuvant (over) treatment being given.

Additional issues influencing the development, choice and use of markers include the impact of legislation (such as *Good Clinical Practice *and *Good Laboratory Practice*) and ethical approval processes on the funding, management, ownership and access to tissue collections and associated clinical data. Academic pressures (particularly the influence in the UK of the Research Assessment Exercise) may be counterproductive to collaborative translational research, which should (but may not be) recognised as of high value.

#### Translational implications

Integrating established and new approaches to prediction and prognosis continues to present challenges. Networks of collaboration (for example, tissue banking and collection of linked patient, tumour and molecular data) employing systems biology (for example, information technology, modelling, identification of key nodes) are still needed for breast cancer research, despite UK and European initiatives. Such developments could speed the development, testing and implementation of new methods of detection and markers of disease behaviour.

We need to recognise the diversity of breast cancer using both traditional and new markers to individualise therapy. Traditionally, trials have classed all women with breast cancer as a single population with a single disease and many have focused on women who have a good prognosis. Future studies should also investigate other groups, such as women least likely to benefit from adjuvant therapy and those with a higher risk of relapse. Targeting breast cancer therapies to those most likely to benefit and avoiding treatment in patients or tumours not responsive could significantly focus benefits on patients likely to respond but also prevent avoidable toxicity.

### 6. Prevention of breast cancer

A summary of the gap analysis for the prevention of breast cancer is given in Table 6.

**Table 6:** Summary of the gap analysis for the prevention of breast cancer

What do we know?	Endocrine chemoprevention for oestrogen-responsive tumours works.
	Key risk factors include mammographic density, post-menopausal weight gain, high-calorie, high-fat diets and lack of exercise.
	Breast screening is effective. MRI screening is more sensitive than mammography for high-risk women
	Epidemiological data suggest weight control, low-fat diet and exercise after diagnosis improves outcome of early breast cancer patients.
What are the gaps?	The long-term effects of chemoprevention of ER positive cancers are unknown.
	Prevention of ER-negative cancers remains a challenge.
	There is a need to understand the target cell for breast cancer prevention.
	Need to improve current risk prediction models by including modifiable risk factors.
	The health beliefs of high-risk and population risk women require exploration.
	The effects of breast screening out with currently targeted groups is not known.
	To define deliverable diet and exercise interventions for the primary and secondary prevention of breast cancer.
	To elucidate the mechanism for breast cancer prevention with energy restriction.
Problems	Accrual and retention of women in prevention trials.
	Better models to research new chemoprevention agents.
	Breast screening lags behind advances in imaging technology.
	Poor uptake to diet and exercise trials after diagnosis.
Translational implications	Better identification of high-risk women would allow chemoprevention to be targeted more effectively.
	Defining optimum screening methods will ensure more effective use of limited NHS resources.
	The development of energy-restriction mimetics for breast cancer prevention.
	Optimal diet and exercise interventions could improve quality of life and outcome for women with breast cancer.
Recommendations	Improve breast cancer risk prediction models.
	Encourage transdisciplinary input to prevention trials (for example, geneticists, epidemiologists, nutritionists, psychologists and clinicians) to study the psychosocial, compliance and genetic aspects of prevention.
	Establish the potential benefits of diet and exercise post-diagnosis on outcome and quality of life for breast cancer patients.

#### Prediction and primary prevention of breast cancer

##### What do we know?

Several large-scale randomised controlled trials show that endocrine chemoprevention for oestrogen-responsive tumours works [89,90]. Breast cancer prevention programmes need to target women at highest risk. Risk prediction programmes predict how many cancers will occur in a population [91], but we need to improve their specificity. We currently need to treat 50 'high-risk' women to prevent one cancer.

Observational studies define mammographic density as one of the strongest risk factors for breast cancer (relative risk 4.64 (3.64 to 5.91) >75% relative to <5% density) [92]. Expert opinion suggests that 20% to 80% of risk is linked to diet [93]. Measurement error with accepted diet assessment methods (food frequency questionnaires) may, however, fail to correctly identify dietary risk factors. For example, recent studies using food diaries, but not food frequency questionnaires, have linked dietary fat to risk [94,95]. Gene-environment (diet) interactions are a further complexity. Failure to consider genotype may mask significant associations with diet; likewise, the effects of genetic polymorphisms may be detectable only with specific dietary exposures (that is, the effect of dietary isoflavone intake on levels of sex-hormone binding globulin among women with the N-variant of the *D356N *gene) [96].

Observational data link weight gain to the risk of post-menopausal breast cancer and lack of exercise to both pre- and post-menopausal breast cancer. Weight loss before and after the menopause reduces post-menopausal risk by up to 40% [97,98]. The observational nature of these studies means that the independent effects of energy restriction, exercise or reductions in adiposity on risk reduction are not known.

##### What are the gaps?

The long-term effects of chemoprevention for ER-positive tumours (beyond five-year study periods) are not known, while preventing ER-negative tumours remains a challenge. Key research areas to inform the development of chemoprevention agents include understanding the target cell (stem or ER-negative cells) and the target lesion in the breast (hyperplasia, atypical ductal hyperplasia, hyperplastic enlarged lobular unit or DCIS) and why the ER becomes deregulated. We need short-term intervention studies of preventive agents and strategies, using pre- and post-intervention biopsies and fine needle aspirates (Ki67, ER).

Risk prediction models need to be improved by including modifiable risk factors, such as mammographic density and lifestyle factors. Key problems that need to be resolved regarding breast density are what mammographic breast density actually measures and whether it is absolute or percentage breast density that is most related to risk. Prospective studies of breast density (using standardised methods) and demographic (body size and drug history) data are required to elucidate factors (that is, age and lifestyle factors, and weight) that modify breast density, and whether these modify the relationship between density and risk.

Elucidating the role of diet in the aetiology of breast cancer requires prospective study of diet and genetic variation, using sensitive dietary assessment methods (food diaries). The relative effects of energy restriction, exercise and reduced adiposity on risk are unlikely to be determined from epidemiology and need to be unravelled by short-term controlled biomarker trials. In addition, the role of adipose tissue and its secretory products (oestrogen and adipokines) in breast cancer development and progression need to be examined from laboratory studies. Since weight loss is difficult to achieve and maintain, effective weight-loss strategies and the benefits of energy restriction mimetic for cancer prevention need to be determined [99]. Psychosocial research is required to explore the health beliefs of high-risk and population-risk women, and to determine their potential interest in lifestyle or pharmacological strategies for breast cancer prevention.

##### What are the problems?

Accrual and retention in prevention trials is a problem; there is currently around a 10% uptake and a 30% dropout rate. Better risk prediction would enable recruitment to be targeted at women with higher levels of risk and motivation. We also need to make the public aware of the cancer prevention message (as with the *Know Your Gail Score *campaign in the USA). We need more diverse recruitment to studies in terms of age and ethnicity.

Transdisciplinary input is needed within prevention trials (for example, geneticists, epidemiologists, nutritionists, psychologists and clinicians) to study the psychosocial, compliance and genetic aspects of prevention.

We also need better models to research new chemoprevention agents, such as stem cell progenitor assays and human breast tissue in nude mice, and better surrogate markers of breast cancer risk. Further mammographic density research requires standardised methods of measurement that need to keep pace with technology in breast screening (for example, digitised mammography and computer-aided detection programmes).

Funding is an issue because prevention and psychosocial research has not been a high priority. Intervention studies are labour intensive and often require more funding than basic science research or than that currently available in project grants.

##### Translational implications

Identifying women who are at high risk of developing breast cancer would enable clinicians to target chemoprevention more effectively. Likewise, unravelling the links between diet and lifestyle, genetic variation and risk would enable us to target lifestyle cancer prevention strategies at the women who would benefit most. Elucidating how lifestyle factors influence risk would underpin an evidence-based lifestyle cancer prevention message, and may enable us to develop drugs that mimic their effects (that is, energy restriction mimetics).

#### Breast screening

##### What do we know?

Breast screening using mammography reduces mortality from breast cancer in women older than 50 (see [100]), and there is limited evidence among women at high risk of breast cancer (lifetime risk of more than 1:6) aged 40 or older [101-103]. Contrast-enhanced magnetic resonance imaging (MRI) is more sensitive than mammography for detecting cancer in high-risk women [104].

##### What are the gaps?

We do not know the benefits of screening women younger than 40 or whether MRI reduces mortality from breast cancer among high-risk women. We also need to determine whether the newer 3 Tesla MRI machines are more sensitive than the 1.0 to 1.5 Tesla MRI machines that were used in earlier studies. We need to conduct further studies to define the best surveillance method for women with dense breasts, women at high risk and women who have had breast cancer, and consider the potential for using other techniques, such as ultrasound or infrared imaging, to screen these women.

##### What are the problems?

Research needs to keep pace with improving imaging technology. There are few data on the uptake of women older than 70 to the National Breast Screening Programme. The recent decline in attendance for screening in the general (eligible) population suggests we need to explore women's health beliefs regarding breast screening. For example, does attending screening negate women's interest in prevention strategies because many women perceive screening as prevention?

##### Translational implications

Defining optimum screening methods in different populations may save lives and ensure more effective use of limited resources within the NHS.

#### The importance of diet and exercise after a diagnosis of early breast cancer

##### What do we know?

Weight, diet and exercise all have an impact on outcome after a diagnosis of breast cancer. Many breast cancer patients are overweight at the time of diagnosis, while many more gain weight after diagnosis, which may increase their risk of dying from breast cancer [105,106] or from weight-related co-morbidities [107] (for example, cardiovascular disease and other cancers). Recent data from a randomised controlled trial (the Women's Intervention Nutrition Study (WINS)) in the USA reported a 24% improved relapse-free survival over five years among post-menopausal breast cancer patients following a low-fat diet (commenced within the first year of diagnosis), compared with the group receiving usual care [108]. Since weight control occurred alongside this low-fat diet it is unclear whether low fat or weight control was linked with improved survival in this study.

The feasibility of following a low-fat diet among breast cancer patients has been piloted in the WINS UK study, where 50% of breast cancer patients achieved a low-fat intake (less than 20% energy from fat) [108]. Observational data link exercise after diagnosis (more than 3 hours per week) with improved survival [109]. A recent systematic review linked exercise during and after treatment to improved physical function and reduced fatigue, while benefits in quality of life were mainly seen in the post-treatment phase [110].

##### What are the gaps?

The data on weight and outcome have come from historic cohorts. The effect of weight and weight gain on outcome alongside current adjuvant therapies needs to be determined. We need to define deliverable diet and exercise interventions after diagnosis, and the optimum timing and mode of delivery for interventions need to be derived from high-quality randomised controlled trials both during and after treatment.

Improved breast cancer survival among recent cohorts shifts the focus to co-morbidities. However, there are few data on the prevalence of co-morbidities or their effect on outcome.

##### What are the problems?

There has been a poor uptake to diet and exercise trials post-diagnosis. More patient-centred qualitative studies are needed to establish preferences along with barriers and motivators to changing behaviour after diagnosis. The feasibility of delivering diet and exercise interventions post diagnosis within the NHS is a potential concern. Healthcare professionals need to 'buy in' as diet and exercise traditionally are not priorities among breast cancer teams.

We also need to consider potential funding, attitudes and work pressures of healthcare professionals, to decide who is best placed to deliver interventions. Good biomarkers of prognosis and measurements of quality of life among breast cancer patients need to be defined so we can evaluate the effect of diet and exercise interventions in trials.

##### Translational implications

Optimum diet and exercise interventions may improve outcomes and quality of life for people with breast cancer.

### 7. Psychosocial aspects of breast cancer

A summary of the gap analysis for the psychosocial aspects of breast cancer is given in Table 7.

**Table 7:** Summary of the gap analysis for the psychosocial aspects of breast cancer

What do we know?	There are psychosocial effects of genetic testing, prophylactic mastectomy and breast screening.
	Descriptive studies of the experiences of breast cancer patients using quantitative and qualitative methods show women still experience psychosocial distress despite improvements in treatment and prognosis.
	Psychosocial interventions have been shown to benefit women, including those identified as experiencing high levels of distress.
What are the gaps?	Evaluation of decision aids for risk management and the choice of preventative surgery amongst high-risk women.
	Ways of effectively communicating information and aiding patient treatment decision-making.
	Defining patient experiences in early, chronic and end stage breast cancer.
	Limited research into co-morbidities amongst breast cancer patients.
	Experiences of ethnic minority populations and older women.
	The need to develop and evaluate appropriate psychosocial interventions for high-risk women and those diagnosed as having breast cancer.
	Use of psychological theories in behaviour change that could enhance compliance to lifestyle and chemoprevention trials.
Problems	The need for the long-term follow-up in psychosocial research.
	Barriers to the uptake of research findings.
Translational implications	Direct improvement in the experience of patients, their families and those at increased risk.
Recommendations	Develop and rigorously evaluate appropriate psychosocial interventions.
	Encourage cross-speciality collaboration to incorporate psychosocial issues and psychological theory (for example, psychological theories in relation to behaviour change are relevant to those researching prevention with diet and exercise or chemoprevention).
	Ensure research gives greater attention to all stages of breast cancer and that the needs of older women and those from a range of ethnic groups are included.

#### Primary prevention of breast cancer: psychosocial aspects of risk and prevention

Genetic testing offers psychosocial benefits for many women at high risk of developing breast cancer, but patients' perceptions of risk are often inaccurate and while many methods have been used to communicate information about risk, they do not always improve patients' understanding [111].

##### What do we know?

Research into the psychosocial impact of bilateral risk-reducing mastectomy is limited, but suggests that while women may be satisfied with their decision to undergo surgery, they may report dissatisfaction with their body image afterwards. Most women are less worried about cancer after surgery, but this may be because they overestimate their risk of developing breast cancer. They need to understand their true risk before considering mastectomy [112]. There is good evidence that counselling can improve the accuracy of perceived risk, but to a varied extent.

Breast screening programmes for women at increased risk do not increase anxiety in women who do not require further investigation, but women who are referred for further investigations are evidently more anxious [113].

##### What are the gaps?

Exploring issues around cancer genetics is paramount, such as follow-up studies of carriers who have or have not undergone risk-reducing surgery, and their partners. Research is needed into the impact of different modes of risk counselling on perceived risk accuracy, healthcare behaviour and uptake of risk-management options. We also need to develop and evaluate decision aids for risk management and choice of surgery. Research also needs to examine further the effect of communicating risk among partners and families of women diagnosed or identified as having an increased chance of developing the disease.

#### After diagnosis: psychosocial aspects for patients with early and advanced disease

##### What do we know?

Areas currently well supported include descriptive studies of the experiences of patients, carers, family and partners [114,115]; the comparative impact of mastectomy versus conservative surgery [116] and of breast reconstruction [117]; aspects of doctor-patient communication [118,119]; recognition of the need to include quality-of-life evaluation in cancer treatment trials; return to work after treatment [120] and potential benefits of exercise on quality of life [121]. This research has fruitfully employed both quantitative and qualitative methods to demonstrate that biomedical developments are not a panacea for the psychosocial distress associated with the disease. Psychosocial interventions have been shown to benefit distressed patients, but are of less value in patients unselected on grounds of psychological functioning [122].

##### What are the gaps?

It is imperative that psychosocial research keeps pace with developments in biomedical treatment and care, including the surgical and adjuvant options that are available (for example, new surgical procedures for reconstructive surgery and developments in hormone therapy), ways of effectively communicating information about these options and aiding patient decision-making where appropriate. An evaluation of self-help strategies is warranted and we also need to broaden the research agenda beyond psychological distress to include interventions for body image and sexual problems.

Research has identified patients' priorities for future research, namely the impact of cancer on life (how to live with cancer), risk factors and causes, early detection and prevention [123]. Psychosocial research currently focuses on early and end-stage breast cancer, yet most patients are not under the care of a palliative team and have to live with their disease and the symptoms of adjuvant treatment (for example, bone pain, neuropathy, physical changes to appearance) as a chronic condition. The experiences of patients in this situation are a significant gap in our knowledge. Future research therefore needs to consider breast cancer in terms of three periods, early, chronic and end, and to examine the specific needs of patients associated with each stage [123]. The specific support needs of both breast cancer patients and their families following the diagnosis of recurrence also warrants further research [124].

There is only limited research into co-morbidities, yet cancer is rarely experienced in isolation and many patients are faced with the effects of the disease alongside other, often chronic, health conditions. We need to investigate how to provide appropriate psychosocial care for patients in this situation.

#### Topics and issues relevant to both preventative and post-diagnosis research

##### What do we know?

There is now a significant body of research into the many psychosocial issues surrounding breast cancer. This research has employed a range of research methods to explore patients' experiences both before and after diagnosis. Although the psychosocial impact of breast cancer diagnosis and treatment has been increasingly recognised by researchers and clinicians in recent years, there is still great potential for psychosocial research to inform the provision of care and improve patients' experiences.

##### What are the gaps?

Existing research into both preventative and post-diagnosis issues is predominantly focused on the experiences of white women in the UK and USA with a fluent understanding of English. We urgently need additional research into the experiences of breast cancer patients from a broad range of ethnic minority populations and of those who do not fully understand English so that tailored care can be provided. Similarly, the experiences of older women are under-represented.

While we have identified the nature of many psychosocial issues faced by patients, there is a pressing need to develop and rigorously evaluate appropriate psychosocial interventions. These should include tools to help patients make decisions, optimal methods for communicating risk, ways of dealing with the effects of treatment and return to work and support for healthcare staff at risk of burnout. Research in this area can identify the most effective means of delivering cost-effective, accessible interventions (for example, timing of interventions, roles of healthcare professionals in delivering interventions, comparing group and individual interventions, and using developments in technology for providing information and decision-making).

Cross-specialty collaboration in breast cancer research that incorporates psychological theory should be a priority. For example, psychological theories in relation to behaviour change could enhance research into diet, exercise and chemoprevention interventions so that factors influencing uptake and adherence are examined.

##### What are the problems?

A number of issues relating to the methods currently employed in psychosocial research should be addressed. These include a need for longer-term follow-up of the impact of surgical procedures and risk counselling, to develop appropriate valid and reliable measures, to consult patients about research priorities and to involve a wider range of research participants (see above). The limited funding available for psychosocial research is a problem and limits the duration of follow-up and prospective studies, especially for trials of psychological interventions.

We must identify and address the barriers to the uptake of research findings, for example by continuing to raise awareness of the importance of psychosocial research and interventions and the potential role that all healthcare staff in both primary and secondary care can play in providing psychosocial care [125]. This is important because the essential role played by specialist nurses in providing psychosocial care and the facilitation of research in this area is often undervalued in the NHS.

##### Translational implications

Further rigorous research in this area could directly improve the experience of patients, their families and those at increased risk of breast cancer because their psychosocial needs would be more appropriately and effectively met at all stages of their cancer journey.

## Conclusion

Research into the pathophysiology, detection, treatment, prevention and psychosocial aspects of breast cancer has produced a wealth of knowledge and has led to substantial improvements in the care of people with breast cancer or at high risk of developing the disease.

This analysis has identified numerous gaps that, if resolved, could have a substantial impact on the diagnosis, management and prevention of breast cancer. For progress to occur, the gap analysis panel has made three key recommendations for each of the seven research areas (Table 8). While recognising the barriers to achieving these aims, the Breast Cancer Campaign urges researchers and funding bodies worldwide to target their resources at these priority areas.

**Table 8:** Gap analysis recommendations and future directions

Generic needs	Improved preclinical models.
	Access to appropriate and annotated clinical material.
	Cross-disciplinary working.
1. Genetics of breast cancer	Encourage development of research techniques to allow integrated analysis of sequence-level, epigenetic and large-scale somatic changes.
	Engage in national initiatives for activities such as high-throughput re-sequencing and UK controls.
	Encourage research involving intermediate phenotypes.
2. Initiation of breast cancer	Develop three-dimensional cell culture models, containing multiple cell types, which reflect the tissue architecture of the normal and diseased breast.
	Generate better animal models, particularly for ER-positive tumours, in which gene expression can be manipulated in all cell types of the mammary gland and will not be altered by transdifferentiation or dedifferentiation.
	Gain a greater understanding of the genetic changes that occur within atypias and DCIS.
3. Progression of breast cancer	Improve preclinical models, research reagents and technologies (including imaging).
	Enhance access to appropriate clinical material, including sequential samples obtained during treatments extending to new agents.
	Consider genetic signature/specific genetic lesions when exploring progression biology and designing clinical trials.
4. Therapies and targets in breast cancer	Build resources through the high-quality, uniform, multicentre collection of clinical material from breast cancer patients before and during treatment (including neoadjuvant studies), including samples of primary tumours as well as metastatic deposits.
	Develop methods for easy, reproducible monitoring of response to and development of resistance to therapy, as well as early disease progression.
	Increase research efforts into the role of the tumour microenvironment and the immune system in the development and treatment of breast cancer.
5. Disease markers in breast cancer	Design innovative trials and translational studies to develop and evaluate predictive and prognostic markers.
	Develop close multidisciplinary collaboration with high-quality histopathology and rigorous scientific assessments to validate new markers important for patient outcome.
	Identify robust markers of resistance or sensitivity to therapy that can be applied across the spectrum of breast disease from screen-detected to metastatic breast cancer.
6. Prevention of breast cancer	Improve breast cancer risk prediction models.
	Encourage transdisciplinary input to prevention trials (for example, geneticists, epidemiologists, nutritionists, psychologists and clinicians) to study the psychosocial, compliance and genetic aspects of prevention.
	Establish the potential benefits of diet and exercise post-diagnosis on outcome and quality of life for breast cancer patients.
7. Psychosocial aspects of breast cancer	Develop and rigorously evaluate appropriate psychosocial interventions.
	Encourage cross-speciality collaboration to incorporate psychosocial issues and psychological theory (for example, psychological theories in relation to behaviour change are relevant to those researching preventative lifestyles including diet and exercise).
	Ensure research gives greater attention to all stages of breast cancer and that the needs of older women and those from a range of ethnic groups are included.

## Abbreviations

COX-2 = cyclo-oxygenase-2; DCIS = ductal carcinoma in situ; DDR = DNA damage response; DNA = deoxyribonucleic acid; EGFR = epidermal growth factor receptor; ER = oestrogen receptor; MRI = magnetic resonance imaging; mRNA = messenger ribonucleic acid; NHS = National Health Service; RNA = ribonucleic acid; RTK = receptor tyrosine kinase; SNP = single nucleotide polymorphism; TMA = tissue microarray; WINS = Women's Intervention Nutrition Study.

## Competing interests

AT, JG, DH, MH and IH are current members of Breast Cancer Campaign's Scientific Advisory Board. AT, KB, AC, JG, DH, MH, IH and CS are current Breast Cancer Campaign grant holders.

## Authors' contributions

AT, KB, AC, JG, DH, MH and IH designed the meeting format. All authors read and approved the final manuscript.
